# The Predictive Value of Neutrophil‐To‐Lymphocyte Ratio (NLR) for Response to Neoadjuvant Immunochemotherapy in Resectable IIA–IIIB Non‐Small Cell Lung Cancer in Real‐World Practice

**DOI:** 10.1111/1759-7714.70315

**Published:** 2026-07-02

**Authors:** Di Meng, Jiacong Liu, Gaoqi Ye, Linhai Zhu, Xuhua Huang, Xiayi Lv, Jian Hu

**Affiliations:** ^1^ Department of Thoracic Surgery, the First Affiliated Hospital Zhejiang University School of Medicine Hangzhou China; ^2^ Key Laboratory of Clinical Evaluation Technology for Medical Device of Zhejiang Province Hangzhou China

**Keywords:** neoadjuvant immunochemotherapy, neutrophil‐to‐lymphocyte ratio (NLR), non‐small‐cell lung cancer (NSCLC), predictive value, real‐world practice

## Abstract

**Objective:**

A significant clinical challenge remains the identification of non‐small cell lung cancer (NSCLC) patients who will derive maximum benefit from neoadjuvant immunochemotherapy. The neutrophil‐to‐lymphocyte ratio (NLR) may offer critical insights into the host's capacity to mount an effective anti‐tumor response.

**Methods:**

This retrospective study consecutively included patients with stage IIA–IIIB NSCLC who received 2–4 cycles of platinum‐based immunochemotherapy prior to planned resection at the Department of Thoracic Surgery, the First Affiliated Hospital, Zhejiang University School of Medicine from 2017 to 2022.

**Results:**

A total of 317 patients were included in our study and divided into two groups according to the median baseline NLR (3.27) of the whole population: low NLR (< 3.27) group (160 patients) and high NLR (≥ 3.27) group (157 patients). The rate of MPR in the low NLR group was significantly higher than that in the high NLR group (58.3% vs. 38.5%, *p* = 0.008). The median PFS in the low NLR group was not reached and 21.6 months (95% confidence interval [CI], 16.9 to 26.3) in the high NLR group (hazard ratio [HR] for disease progression, disease recurrence, or death, 0.431; 95% CI, 0.312 to 0.594; *p* < 0.001). The median OS in the low NLR group was not reached and 33.2 months (95% CI, 18.8 to 47.6) in the high NLR group (HR for death, 0.416; 95% CI, 0.295 to 0.586; *p* < 0.001).

**Conclusions:**

A low pre‐treatment NLR is a predictive marker of enhanced pathological response and improved survival outcomes in IIA–IIIB NSCLC patients undergoing neoadjuvant immunochemotherapy.

## Graphical abstract

A low pre‐treatment NLR predicts better pathological response, higher surgical resection rate, and superior survival outcomes in patients with stage IIA–IIIB NSCLC receiving neoadjuvant immunochemotherapy, without compromising safety. NLR may serve as a readily available biomarker to guide clinical decision‐making.

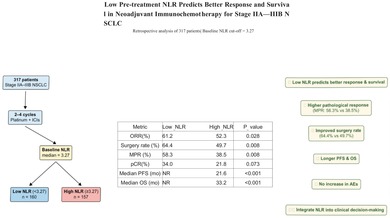

**Abbreviations**
AEs: adverse events
AJCC: American Joint Committee on Cancer
CI: confidence interval
CR: complete response
CT: computed tomography
CTCAE: Common Terminology Criteria for Adverse Events
ECOG: Eastern Cooperative Oncology Group
EFS: event‐free survival
HR: hazard ratio
ICIs: immune checkpoint inhibitors
IQR: interquartile range
LUAD: lung adenocarcinoma
LUSC: lung squamous cell carcinoma
MPR: major pathologic response
MRI: magnetic resonance imaging
NA: not acquired
NLR: neutrophil‐to‐lymphocyte ratio
NSCLC: non‐small‐cell lung cancer
ORR: objective response rate
OS: overall survival
pCR: pathologic complete response
PD: progressive disease
PET: positron emission tomography
PFS: progression‐free survival
PR: partial remission
RATS: robot‐assisted thoracoscopic surgery
RECIST 1.1: response evaluation criteria in solid tumor version 1.1
SD: stable disease
TMB: tumor mutational burden
TME: tumor microenvironment
TNM: tumor–node–metastasis
VATS: video‐assisted thoracoscopic surgery

## 1 Introduction

Lung cancer, primarily manifested as NSCLC, poses a formidable challenge to global health, continuing to claim more lives than any other malignancy [1]. The management of resectable NSCLC has been fundamentally transformed by the advent of neoadjuvant immunotherapy. Landmark trials, notably CheckMate 816, established that combining neoadjuvant chemotherapy with immune checkpoint inhibitors (ICIs) significantly enhances pathological complete response (pCR) rates and extends event‐free survival (EFS), thereby creating a new therapeutic paradigm [2]. Despite this progress, a substantial proportion of patients exhibit inherent resistance to these regimens, deriving minimal clinical benefit while being exposed to potential immune‐related adverse events. This clinical heterogeneity underscores an urgent, unmet need for discovering easily measurable biomarkers that can forecast therapeutic efficacy and facilitate personalized treatment strategies [3].

The quest for predictive biomarkers has largely focused on tumor‐intrinsic properties like PD‐L1 expression and tumor mutational burden (TMB). However, their predictive utility remains imperfect, characterized by variable assay standards and considerable heterogeneity within tumors [4]. Consequently, attention has shifted toward host‐related factors, particularly the systemic inflammatory and immunological milieu, which is increasingly recognized as a critical determinant of ICI efficacy. The dynamic interplay between pro‐tumorigenic inflammation and anti‐tumor immunity forms a crucial axis that can either support or hinder treatment success [5].

The tumor microenvironment (TME) plays a pivotal role in cancer progression and therapeutic response. Beyond the tumor cell‐centric view, the host's systemic inflammatory and immune status is increasingly recognized as a key determinant of outcomes [6]. The neutrophil‐to‐lymphocyte ratio (NLR), a readily accessible and cost‐effective biomarker derived from routine complete blood count, serves as a quantitative measure of this systemic balance. It integrates two opposing immune pathways: pro‐tumor inflammation, represented by neutrophils, and anti‐tumor immunity, mediated by lymphocytes [7]. Elevated neutrophil levels can promote tumor angiogenesis, tissue invasion, and suppress adaptive immune responses, while lymphocytopenia reflects an impaired capacity for immune surveillance and cytotoxicity [8]. Consequently, a high NLR embodies a state of heightened systemic inflammation coupled with relative immunosuppression, a milieu conducive to tumor progression and resistance to therapy.

Extensive evidence has corroborated the prognostic value of NLR across various cancer types, including lung cancer [9]. In the metastatic setting, a high baseline NLR is consistently associated with poorer overall survival and reduced benefit from chemotherapy and targeted therapies [10]. Its significance is particularly pronounced in the context of immunotherapy, as the efficacy of ICIs is heavily dependent on a pre‐existing and competent T‐cell lymphocyte population, the very component compromised in a high NLR phenotype [11]. While the prognostic role of NLR is well documented in advanced stages, its predictive utility in the neoadjuvant setting for NSCLC is a subject of intense and ongoing investigation. Emerging data suggest that a low pre‐treatment NLR is significantly associated with better survival outcomes in patients with NSCLC receiving ICIs [12]. However, the integration of this biomarker into clinical decision‐making for neoadjuvant therapy requires further validation and standardization. Therefore, this study aims to conduct a comprehensive evaluation of NLR's predictive significance for both treatment response and long‐term survival in the locally advanced NSCLC patients undergoing neoadjuvant immunochemotherapy.

## 2 Methods

### 2.1 Study Design and Patients

This retrospective analysis was conducted as a single‐center, real‐world investigation at the Department of Thoracic Surgery, the First Affiliated Hospital, Zhejiang University School of Medicine. The study protocol received full approval from the hospital's Clinical Research Ethics Committee (2021 IIT No. 844). We systematically screened consecutive patients with histologically confirmed stage IIA to IIIB (AJCC TNM staging 8th edition) NSCLC who received neoadjuvant immunochemotherapy between 2017 and 2022. Key inclusion criteria were: (I) completion of 2–4 cycles of neoadjuvant immunochemotherapy; (II) availability of complete medical records at our hospital; (III) no history of another active malignancy; (IV) no history of anticancer treatment; (V) no active autoimmune or infectious disease; (VI) no ongoing systemic immunosuppressive therapy; and (VII) no distant metastases.

### 2.2 Procedures

The immunotherapy protocol consisted of ICIs (camrelizumab, nivolumab, sintilimab, tislelizumab, or pembrolizumab) administered at a fixed dose of 200 mg per cycle. The chemotherapy backbone was platinum‐based, utilizing either cisplatin (75 mg/m^2^) or carboplatin (target AUC = 5) in combination with nab‐paclitaxel (260 mg/m^2^). Patients underwent radical resection through one of three approaches: open thoracotomy, video‐assisted thoracoscopic surgery (VATS), or robot‐assisted thoracoscopic surgery (RATS). Systematic lymph node dissection was routinely conducted in accordance with standard surgical principles.

Prior to the initiation of neoadjuvant treatment and subsequent surgical intervention, all enrolled patients completed a comprehensive diagnostic workup. This included thoracic computed tomography (CT), endoscopic ultrasonography, positron emission tomography [PET]–CT, cerebral magnetic resonance imaging [MRI], and abdominal ultrasonography. Throughout the neoadjuvant treatment phase, radiographic assessment of the chest via CT was performed at intervals corresponding to every two treatment cycles. The follow‐up for each participant was terminated only upon the occurrence of death.

The baseline NLR was determined from peripheral blood samples collected in EDTA tubes during routine clinical workup before the first cycle of neoadjuvant therapy. The absolute neutrophil and lymphocyte counts were extracted directly from the automated CBC differential report. The NLR was calculated as the ratio of the absolute neutrophil count to the absolute lymphocyte count. Patients in our study were divided into two groups according to the median baseline NLR of the whole population. Using the cohort median was an exploratory grouping strategy.

### 2.3 Assessments

This study established dual primary endpoints: progression‐free survival (PFS) and overall survival (OS). Secondary outcome measures included objective response rate (ORR), treatment‐emergent adverse events (AEs), and pathological assessments [specifically major pathological response (MPR) and pathologic complete response (pCR)].

PFS was determined as the timeframe extending from neoadjuvant treatment initiation to the initial confirmation of disease progression or mortality from any origin. OS was quantified from therapy commencement to death from any causation. ORR was assessed per response evaluation criteria in solid tumor version 1.1 (RECIST 1.1), with a threshold of ≥ 30% reduction from baseline in the aggregate diameter of all target lesions. AEs' severity was classified using the Common Terminology Criteria for Adverse Events (CTCAE) version 5.0. The MPR was characterized by the presence of no more than 10% residual viable tumor cells in both the primary tumor site and the evaluated lymph nodes following comprehensive histological assessment of surgical specimens. The pCR was characterized by the total eradication of viable tumor cells, with 0% residual tumor identified in the primary tumor site and all examined regional lymph nodes. Two board‐certified pathologists, blinded to preoperative NLR data, conducted independent histological evaluations of all resected specimens using standardized scoring criteria. Any discrepant cases were resolved through a consensus review.

### 2.4 Statistical Analysis

Continuous data were summarized as medians with interquartile ranges (IQR) and analyzed using either the *t*‐test or Wilcoxon test based on distributional assumptions. Categorical variables were reported as frequencies with percentages, with between‐group comparisons performed using Chi‐square or Fisher's exact tests as statistically appropriate. Survival outcomes, including PFS and OS, were evaluated using Kaplan–Meier methodology with between‐group comparisons performed via log‐rank testing. The median follow‐up duration was calculated applying the reverse Kaplan–Meier approach. Statistical significance was defined as a two‐sided *p*‐value below 0.05. All statistical computations were conducted using R statistical software (version 4.1.2).

## 3 Results

### 3.1 Baseline Characteristics

**FIGURE 1:**
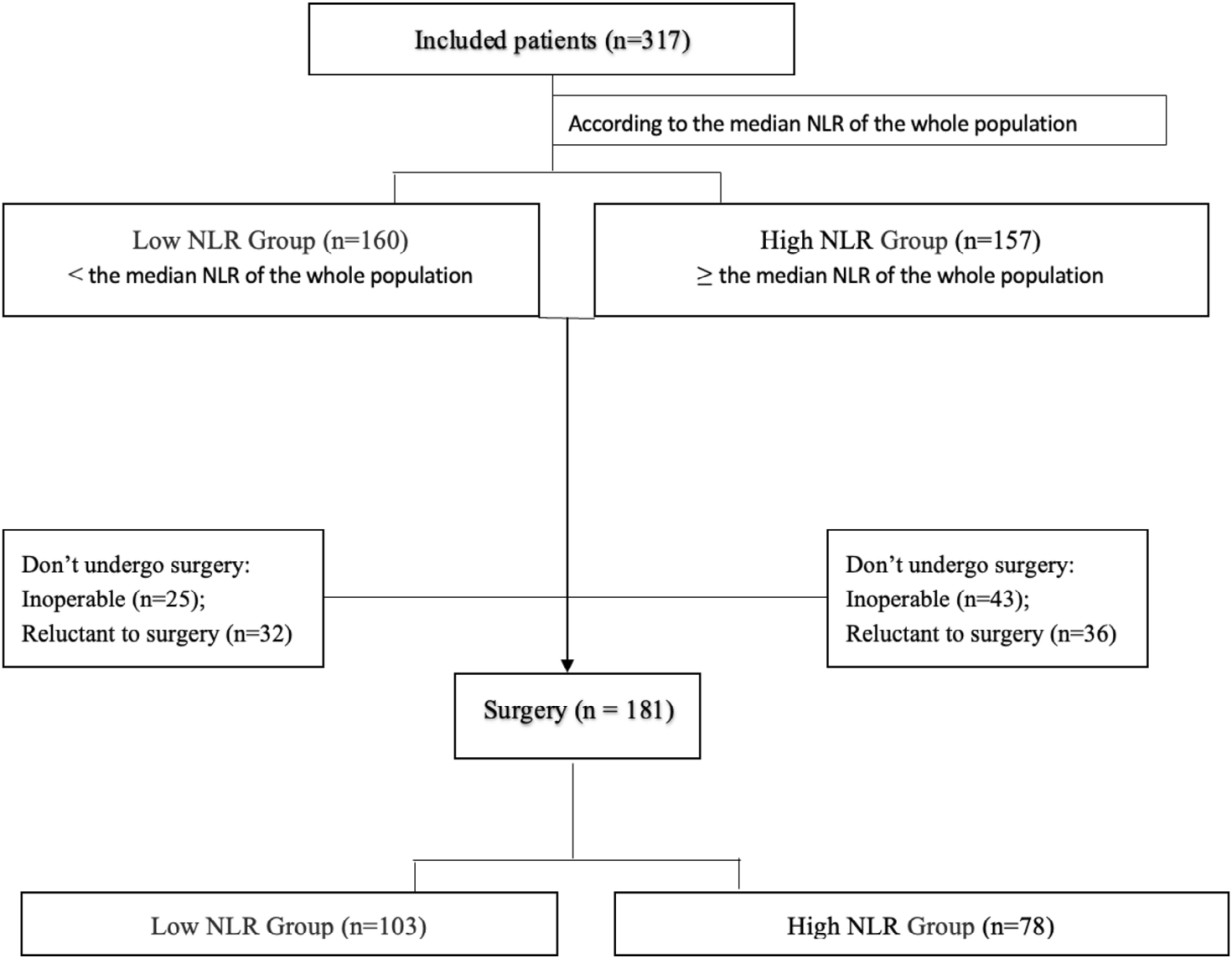
The design flowchart of this study. NLR, neutrophil‐to‐lymphocyte ratio.

A total of 317 patients were included in our study and divided into two groups according to the median baseline NLR (3.27) of the whole population: low NLR (< 3.27) group (160 patients) and high NLR (≥ 3.27) group (157 patients). Baseline characteristics were well balanced between the two cohorts, as summarized in Table 1. Figure 1 illustrated the patient enrollment and study design workflow.

**TABLE 1:** Characteristics at baseline.

Variables	Low NLR, *n* = 160	High NLR, *n* = 157	*p*
Age (years), median age (IQR)	65.0 (59.0–70.0)	65.0 (60.0–70.0)	0.149
Sex, *n* (%)			0.357
Male	143 (89.4)	145 (92.4)	
Female	17 (10.6)	12 (7.6)	
ECOG performance status, *n* (%)			0.697
0	100 (62.5)	102 (65.0)	
1	59 (36.9)	53 (33.8)	
2	1 (0.6)	2 (1.2)	
Smoking status, *n* (%)			0.501
Never	65 (40.6)	58 (36.9)	
Ever	95 (59.4)	99 (63.1)	
Drinking status, *n* (%)			0.097
Never	112 (70.0)	96 (61.1)	
Ever	48 (30.0)	61 (38.9)	
Diabetes mellitus, *n* (%)			0.595
Yes	16 (10.0)	13 (8.3)	
No	144 (90.0)	144 (91.7)	
Hypertension, *n* (%)			0.082
Yes	54 (33.8)	39 (24.8)	
No	106 (66.2)	118 (75.2)	
T stage, *n* (%)			0.557
T1b	9 (5.6)	9 (5.7)	
T1c	19 (11.9)	24 (15.3)	
T2a	31 (19.4)	28 (17.8)	
T2b	27 (16.9)	36 (23.0)	
T3	44 (27.5)	32 (20.4)	
T4	30 (18.8)	28 (17.8)	
N stage, *n* (%)			0.356
N0	8 (5.0)	7 (4.5)	
N1	22 (13.8)	29 (18.5)	
N2	124 (77.5)	110 (70.1)	
N3	6 (3.8)	11 (7.0)	
Clinical stage, *n* (%)			0.368
IIA	9 (5.6)	4 (2.6)	
IIB	17 (10.6)	17 (10.8)	
IIIA	84 (52.5)	76 (48.4)	
IIIB	50 (31.3)	60 (38.2)	
Pathology, *n* (%)			0.138
LUSC	116 (72.5)	125 (79.6)	
LUAD	44 (27.5)	32 (20.4)	
Treatment cycle, *n* (%)			0.364
2	68 (42.5)	55 (35.0)	
3	23 (14.4)	28 (17.8)	
4	69 (43.1)	74 (47.2)	
Immunotherapy regimes, *n* (%)			0.214
Camrelizumab, 200 mg	56 (35.0)	45 (28.7)	
Nivolumab, 200 mg	20 (12.5)	26 (16.6)	
Sintilimab, 200 mg	20 (12.5)	20 (12.7)	
Tislelizumab, 200 mg	23 (14.4)	35 (22.3)	
Pembrolizumab, 200 mg	41 (25.6)	31 (19.7)	

Abbreviations: ECOG, Eastern Cooperative Oncology Group; IQR, interquartile range; LUAD, lung adenocarcinoma; LUSC, lung squamous cell carcinoma; NLR, neutrophil‐to‐lymphocyte ratio.

### 3.2 Response to Neoadjuvant Therapy

**FIGURE 2:**
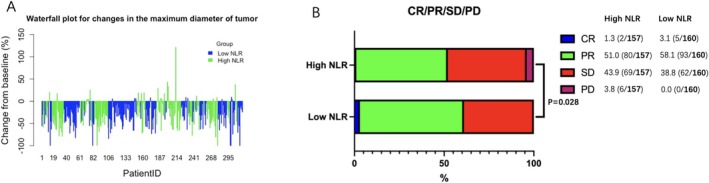
(A) The percentage change in the maximum diameter of the target lesion compared with the baseline tumor size in the whole population (*n* = 317). (B) The distribution condition of clinical response in the whole population (*n* = 317). Clinical response included complete response (CR), partial remission (PR), and stable disease (SD). NLR, neutrophil‐to‐lymphocyte ratio.

As shown in Figure 2A, a substantial decrease in the largest target lesion diameter was documented when compared with baseline measurements. This trend was evident across the majority of patients in both treatment groups. Progressive disease (PD) events were exclusively documented in the high NLR cohort (6 cases), with no PD instances observed in patients with low NLR values (Figure 2B). The ORR in the low NLR and high NLR groups was 61.2% and 52.3% (*p* = 0.028), respectively.

### 3.3 Adverse Events

AEs of neoadjuvant therapy were consistent with established expectations, with no new or unexpected AEs identified in this cohort. As detailed in Table 2, the incidence of AEs was comparable between the low and high NLR groups (85.0% vs. 87.9%, *p* = 0.536). The safety profiles were comparable between the two treatment groups, with no statistically significant differences in AEs incidence. All reported adverse events were effectively managed with appropriate symptomatic intervention and demonstrated prompt resolution.

**TABLE 2:** AEs of neoadjuvant therapy.

Variables	Low NLR, *n* = 160	High NLR, *n* = 157	*p*
Any AEs, *n* (%)	136 (85.0)	138 (87.9)	0.536
Hematologic			
Leukopenia	63 (39.4)	67 (42.7)	0.581
Agranulocytosis	32 (20.0)	32 (20.4)	0.955
Anemia	92 (57.5)	83 (52.9)	0.372
Thrombocytopenia	17 (10.6)	15 (9.6)	0.752
Gastrointestinal			
Nausea	4 (2.5)	3 (1.9)	1.000
Emesis	11 (6.9)	8 (5.1)	0.496
Diarrhea	4 (2.5)	8 (5.1)	0.255
Constipation	28 (17.5)	37 (23.6)	0.19
Hepatic injury	52 (32.5)	68 (43.3)	0.052
Renal injury	7 (4.4)	7 (4.5)	0.981
Immune myocarditis	0 (0.0)	0 (0.0)	NA
Immune pneumonia	3 (1.9)	1 (0.6)	0.623
Skin reaction	27 (16.9)	32 (20.4)	0.438
Hypothyroidism	3 (1.9)	4 (2.5)	0.722
Coagulation disorders	1 (0.6)	4 (2.5)	0.213
Sensory neurotoxicity	6 (3.8)	7 (4.5)	0.759

Abbreviations: AEs, adverse events; NA, not acquired; NLR, neutrophil‐to‐lymphocyte ratio.

### 3.4 Surgical Outcomes and Pathological Response

**FIGURE 3:**
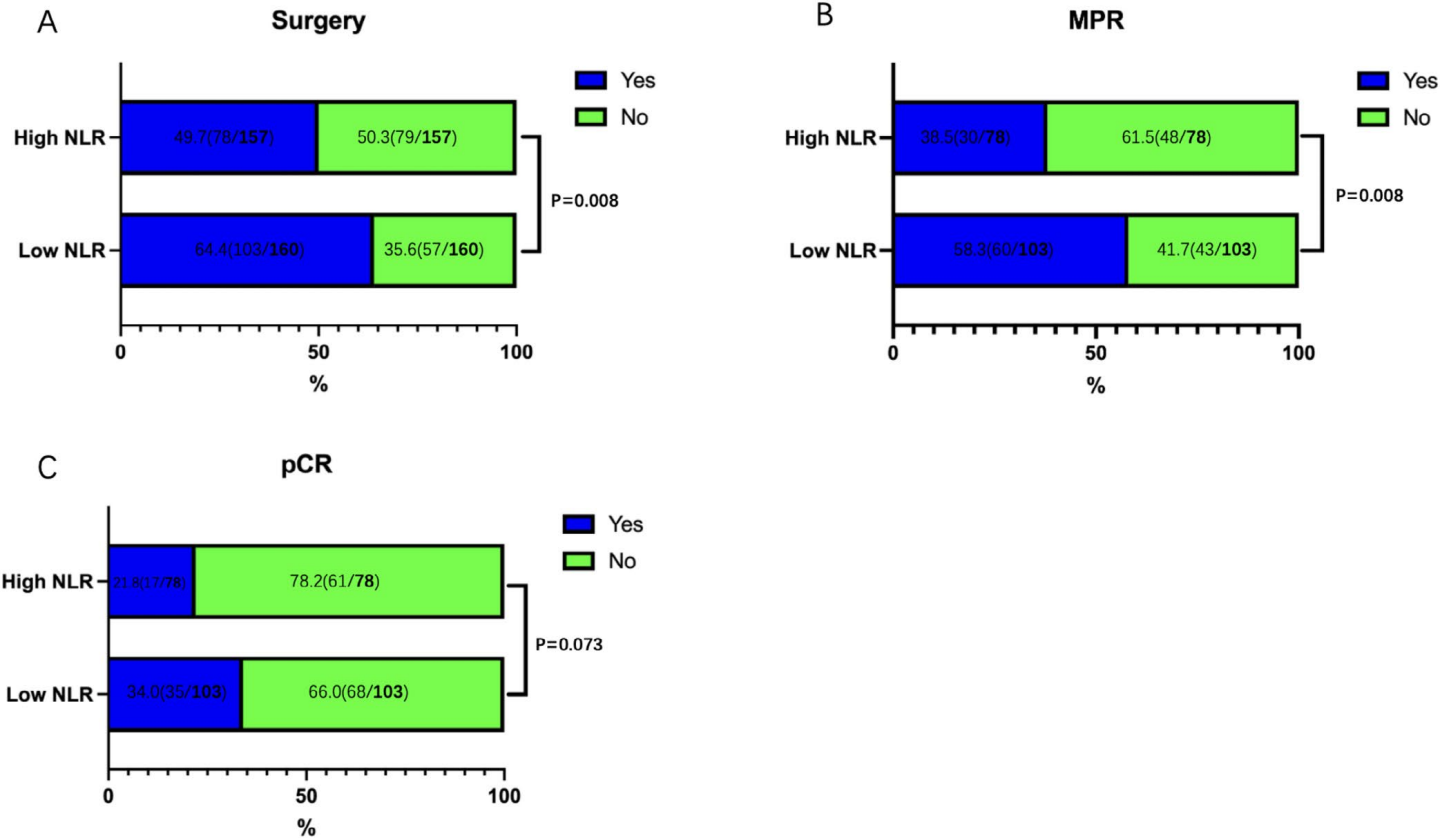
The distribution condition of surgery and pathological response in the surgical population (*n* = 181): (A) Surgery, (B) MPR, (C) pCR. Pathological response included major pathological response (MPR) and pathological complete remission (pCR). NLR, neutrophil‐to‐lymphocyte ratio.

Perioperative outcomes, including surgical results and postoperative complications, are comprehensively presented in Table 3. About 64.4% (103/**160**) in the low NLR group and 49.7% (78/**157**) in the high NLR group eventually underwent surgery (*p* = 0.008) (Figure 3A). The two cohorts demonstrated comparable profiles across multiple perioperative parameters, including treatment‐to‐surgery interval, surgical techniques (approach and methodology), intraoperative metrics (operation duration and blood loss), pathological outcomes (margin status, lymph node yield, tumor grade, and ypTNM staging), as well as postoperative recovery (hospitalization period). The rate of MPR in the low NLR group was significantly higher than that in the high NLR group (58.3% vs. 38.5%, *p* = 0.008) (Figure 3B). The rate of pCR in the low NLR group and high NLR group was 34.0% and 21.8% (*p* = 0.073), respectively (Figure 3C). The overall postoperative complication profiles were comparable between groups. Importantly, no perioperative mortality occurred during the entire study period.

**TABLE 3:** Surgical outcomes.

Variables	Low NLR, *n* = 103	High NLR, *n* = 78	*p*
Time from first treatment to surgery, median (IQR), day	146.5 (110.3–198.0)	146 (109.0–180.0)	0.795
Surgical approach, *n* (%)			0.279
Open	52 (50.5)	48 (61.5)	
VATS	37 (35.9)	18 (23.1)	
RATS	2 (1.9)	1 (1.3)	
VATS‐Open	12 (11.7)	11 (14.1)	
Surgical method, *n* (%)			0.398
Wedge resection	3 (2.9)	4 (5.1)	
Lobectomy	62 (60.2)	41 (52.6)	
Sleeve lobectomy	23 (22.3)	25 (32.1)	
Pneumonectomy	13 (12.6)	8 (10.3)	
Segmental resection	2 (1.9)	0 (0.0)	
Operation time, median (IQR), min	145.0 (108.5–196.0)	146.0 (109.0–180.0)	0.672
Estimated blood loss, median (IQR), mL	50.0 (20.0–100.0)	50.0 (20.0–100.0)	0.825
Resection margin *n* (%)			0.466
R0	100 (97.1)	74 (94.9)	
R1	3 (2.9)	4 (5.1)	
Number of lymph node dissections during surgery, median (IQR), *n*	14.5 (10.0–20.0)	15.0 (10.0–22.0)	0.229
Length of hospital stay, median (IQR), day	12.5 (7.0–17.0)	14.0 (9.0–18.0)	0.706
Postoperative complication, *n* (%)			
Overall	35 (34.0)	32 (41.0)	0.354
Hydropneumothorax	8 (7.8)	13 (16.7)	0.099
Hydrothorax	19 (18.4)	16 (20.5)	0.850
Pneumothorax	7 (6.8)	3 (3.8)	0.519
Chylothorax	0 (0.0)	1 (1.3)	0.431
Bronchial obstruction	1 (1.0)	0 (0.0)	1.000
Pathological grade, *n* (%)			0.343
G1	2 (1.9)	0 (0.0)	
G2	37 (35.9)	32 (41.0)	
G3	35 (34.0)	31 (39.7)	
Unknown	29 (28.2)	15 (19.2)	
Pathological response, *n* (%)			**0.032**
TRG 0	35 (34.0)	17 (21.8)	
TRG 1	25 (24.3)	13 (16.7)	
TRG 2	43 (41.7)	48 (61.5)	
ypTNM stage, *n* (%)			0.186
0	35 (34.0)	17 (21.8)	
IA	25 (24.3)	13 (16.7)	
IB	5 (4.9)	5 (6.4)	
IIA	5 (4.9)	3 (3.8)	
IIB	17 (16.5)	21 (26.9)	
IIIA	15 (14.6)	16 (20.5)	
IIIB	1 (1.0)	3 (3.8)	

*Note:* Bold value indicates the pathological response *P* = 0.032.

Abbreviations: IQR, interquartile range; NLR, neutrophil‐to‐lymphocyte ratio; RATS, robot‐assisted thoracoscopic surgery; VATS, video‐assisted thoracoscopic surgery; ypTNM, post‐neoadjuvant pathologic tumor‐node‐metastasis.

### 3.5 Survival

**FIGURE 4:**
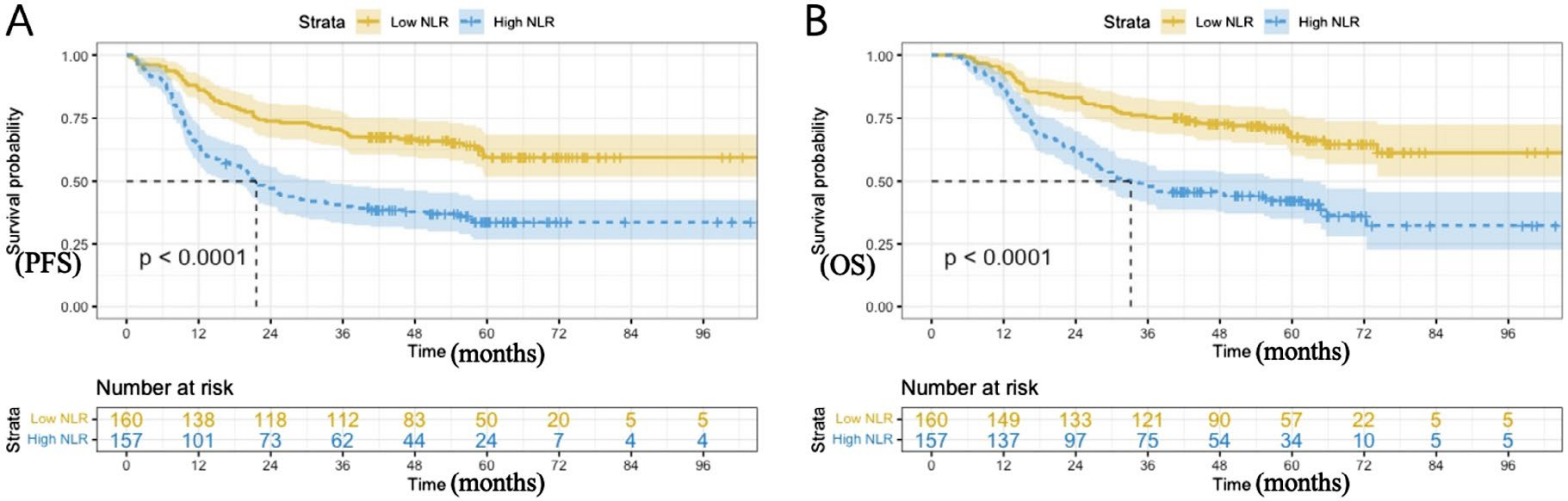
Kaplan Meier curves of PFS (A) and OS (B) between the low NLR group and the high NLR group in the whole population (*n* = 317). NLR, neutrophil‐to‐lymphocyte ratio; PFS, progression‐free survival; OS, overall survival.

As of the September 2025 database lock, complete follow‐up data were available for the entire study population. The low NLR cohort demonstrated a median follow‐up duration of 61.0 months (95% confidence interval [CI], 57.0 to 65.0), compared with 60.2 months (95% CI, 58.1 to 62.3) in the high NLR group. The median PFS in the low NLR group was not reached and 21.6 months (95% CI, 16.9 to 26.3) in the high NLR group (hazard ratio [HR] for disease progression, disease recurrence, or death, 0.431; 95% CI, 0.312 to 0.594; *p* < 0.001) (Figure 4A). The median OS in the low NLR group was not reached and 33.2 months (95% CI, 18.8 to 47.6) in the high NLR group (HR for death, 0.416; 95% CI, 0.295 to 0.586; *p* < 0.001) (Figure 4B).

## 4 Discussion

This study provides compelling and multi‐faceted evidence that baseline NLR, a readily accessible inflammatory biomarker, serves as a powerful and independent biomarker for predicting therapeutic efficacy across the entire spectrum of care for NSCLC patients receiving neoadjuvant therapy. Our findings, which demonstrate a consistent advantage for patients with a low baseline NLR (< 3.27) in terms of tumor response, surgical resectability, pathological regression, and long‐term survival, robustly affirm the critical role of the host's systemic immune‐inflammatory status in modulating treatment outcomes.

The superior ORR observed in the low NLR group (61.2% vs. 52.3%, *p* = 0.028) aligns with the fundamental biology of cancer‐associated inflammation. A low NLR signifies an immune‐conducive state, characterized by restrained neutrophilia and preserved lymphocyte‐mediated cytotoxicity, which is essential for effective tumor cell killing [13]. Our results are consistent with a meta‐analysis by Yang et al. which found that a low NLR was significantly associated with improved ORR in advanced NSCLC patients treated with immune checkpoint inhibitors [12]. The significant disparity in surgical conversion rates (64.4% vs. 49.7%, *p* = 0.008) in our cohort is a critical finding. This suggests that a favorable pre‐treatment immune milieu not only predicts a better initial response but also increases the likelihood of patients achieving a disease state amenable to curative‐intent resection. This finding extends the work of Botta et al. who reported a similar trend where inflammatory markers were linked to resectability following neoadjuvant chemoimmunotherapy [14].

Most notably, the pathological assessment of resected specimens revealed a profound advantage for the low NLR group. The rate of MPR was substantially higher (58.3% vs. 38.5%, *p* = 0.008), and a strong trend toward improved pCR was observed (34.0% vs. 21.8%, *p* = 0.073). MPR and pCR are well‐validated surrogate endpoints for long‐term survival [15]. Our results thus confirm that the systemic immune state, quantified by NLR, is intricately linked to the fundamental biological process of achieving a deep pathological response, which is the ultimate goal of neoadjuvant therapy. Our data corroborate and significantly extend the observations from a smaller study by Russo et al. which identified a correlation between baseline inflammatory markers and pathological response in a cohort receiving neoadjuvant chemotherapy [16]. The magnitude of the benefit we observed in the context of modern neoadjuvant regimens underscores the enduring and perhaps amplified relevance of NLR in the immunotherapy era.

The most striking evidence of the prognostic value of NLR comes from the survival analysis. The low NLR group experienced a remarkable reduction in the risk of disease progression or death (HR 0.431, *p* < 0.001), with a median PFS that was not reached compared to 21.6 months in the high NLR group. Similarly, the risk of death was reduced (HR 0.416, *p* < 0.001), with a median OS not reached versus 33.2 months. These robust hazard ratios underscore NLR's power as an independent prognostic factor, consistent with previous meta‐analyses in various cancer types [9]. The convergence of superior response, higher resection rates, deeper pathological regression, and vastly improved survival in the low NLR group paints a coherent picture of its pivotal role.

It is crucial to highlight that the comparable incidence of AEs between the two groups (15.5% vs. 21.3%, *p* = 0.536) indicates that the superior efficacy in the low NLR cohort was not attained at the expense of increased treatment‐related toxicity. This finding is consistent with the understanding that NLR reflects the host's baseline state rather than being a direct cause of toxicity, thereby affirming its role as a pure efficacy biomarker [11].

Our study has limitations. Its single‐center, retrospective nature necessitates validation in larger, prospective cohorts, potential treatment heterogeneity (different immune checkpoint inhibitors). Furthermore, the precise biological mechanisms linking high NLR to therapy resistance, while likely involving myeloid‐derived suppressor cell activity and T‐cell suppression [6], warrant further mechanistic investigation. And using the cohort median is acceptable in an exploratory analysis, but it limits clinical generalizability. Moreover, the pathological comparisons between NLR groups are susceptible to selection bias, as non‐surgical patients (who had a higher disease progression rate and were more common in the high NLR group) were excluded. Our reported MPR and pCR results may therefore overestimate the treatment effect in the high NLR group. We have explicitly acknowledged this bias as a major limitation.

In conclusion, a low pre‐treatment NLR is a predictive marker of enhanced pathological response and improved survival outcomes in IIA–IIIB NSCLC patients undergoing neoadjuvant immunochemotherapy. NLR emerges as a simple, cost‐effective, and powerful integrative biomarker that stratifies NSCLC patients undergoing neoadjuvant therapy into distinct prognostic groups. It reliably predicts the likelihood of radiographic response, surgical resectability, pathological regression, and ultimately, long‐term survival. Integrating NLR into clinical decision‐making could help identify patients with high NLR who are at high risk of treatment failure and may benefit from more intensive or novel therapeutic strategies, thereby personalizing the management of locally advanced NSCLC. Our results suggest that NLR may be a potential prognostic marker in this setting, but the conclusions are limited by the retrospective, single‐center design. Prospective validation is required before clinical application.

## Author Contributions

**Di Meng:** conceptualization, data curation, formal analysis, investigation, visualization, methodology, writing – original draft. **Jiacong Liu:** conceptualization, data curation, formal analysis, investigation, visualization, methodology, writing – original draft. **Gaoqi Ye:** conceptualization, investigation, visualization, methodology. **Linhai Zhu:** conceptualization, investigation, visualization, methodology. **Xuhua Huang:** conceptualization, investigation, visualization, methodology. **Xiayi Lv:** conceptualization, investigation, resources, supervision, validation. **Jian Hu:** conceptualization, funding acquisition, investigation, resources, supervision, validation, writing – review and editing.

## Funding

This research was supported by the Key Research and Development Projects of “Vanguard” and “Leading Goose” in Zhejiang Province (2025C02095); the National Key Research and Development Program of China (2022YFC2407303); Research Center for Lung Tumor Diagnosis and Treatment of Zhejiang Province (JBZX‐202007).

## Ethics Statement

This trial was approved by the Clinical Research Ethics Committee of the First Affiliated Hospital, Zhejiang University School of Medicine (2021 IIT No. 844), and done in accordance with the Declaration of Helsinki (as revised in 2013) and Good Clinical Practice Guidelines. Written informed consent was obtained from patients so that we could acquire and use required information from their medical record in our hospital.

## Consent

The authors have nothing to report.

## Conflicts of Interest

The authors declare no conflicts of interest.

## Data Availability

The data that support the findings of this study are available on request from the corresponding author. The data are not publicly available due to privacy or ethical restrictions.
